# Supplementary material for: Ultralow-temperature-driven water-based sorption refrigeration enabled by low-cost zeolite-like porous aluminophosphate
Source: Nat Commun. 2022 Jan 11;13:193. doi: 10.1038/s41467-021-27883-4 (PMC8752593; doi:10.1038/s41467-021-27883-4)
Supplement: Supplementary file 1 — Supplementary Information [file 41467_2021_27883_MOESM1_ESM.pdf]

Supplementary Information

for

Ultralow-temperature-driven water-based sorption refrigeration enabled by low-cost zeolite-like porous aluminophosphate

Zhangli Liu[1,3,†], Jiaxing Xu[2,†,], Min Xu[1,3,4*†], Caifeng Huang[1,4], , Ruzhu Wang[2] ,

Tingxian Li[2,*],& Xiulan Huai[1,3,4*]

1. Institute of Engineering Thermophysics, Chinese Academy of Sciences, Beijing 100190, China.
2. Research Center of Solar Power & Refrigeration, School of Mechanical Engineering, Shanghai Jiao Tong University, Shanghai 200240, China.
3. University of Chinese Academy of Sciences, Beijing, 100049, China
4. Nanjing Institute of Future Energy System, Nanjing, 211135, China

† These authors contributed equally to this work

* E-mail: xumin@iet.cn; Litx@sjtu.edu.cn; hxl@iet.cn;

Supplementary Note 1.

Principle and description of typical adsorption heat transfer system.

The working principle of typical adsorption heat transfer system in view of limit thermodynamics can be described by a Carnot cycle, consisting of three temperatures related to evaporation, condensation, and regeneration. The operation of water-sorption driven refrigerators and heat pumps is based on the ability of porous solids as the water adsorbent to adsorb water vapour when at low temperature and to desorb it when heated. The basic configuration for adsorption-driven heat pump and refrigerators cycles is illustrated in **Supplementary Fig. 1a**, including two adsorption chambers to ensure continuous operation. The AHP/ADC cycle consists of four steps, two for adsorption and two for desorption. These steps are briefly explained with the aid of the cycle diagram (**Supplementary Fig. 1b**), starting from a fully saturated adsorbent (**Supplementary Fig. 1b**, point I).

Step 1, Isosteric heating (I-II): During this stage, the adsorption chamber is separated from the evaporator and condenser and no working fluid is desorbed. The saturated adsorbent (W_{max}) requires desorption of working fluid or regeneration. Before working fluid can be released to the condenser, pressure needs to be increased from P_{ev} to P_{con}, which is realized by heating the adsorbent from T_1 to T_2.

Step 2, Isobaric desorption (II−III): Adsorbent heating is continued. Because the adsorption chamber is connected to the condenser in this stage, working fluid is allowed to desorb and no further pressure increase occurs. This process is stopped when

desorption temperature (T_{des}) is reached and the adsorbent loading is minimal (W_{min}), The desorbed working fluid (W_{max}-W_{min}) is condensed, releasing condensation heat (Q_{con}) to the environment.

Step 3, Isosteric cooling (III-IV): The adsorption chamber is isolated from the evaporator and condenser. Before adsorbent regenerated can be used for adsorption, the pressure needs to be reduced to P_{ev} by cooling adsorption chamber from T_{des} to T_3.

Step 4, Isobaric adsorption (IV–I): Cooling is continued. Because the adsorbent vessel is connected to the evaporator in this stage, working fluid is allowed to adsorb and no further pressure decrease occurs. This process is stopped when T_1 is reached and the adsorbent loading is maximal again (W_{max}). The adsorbed working fluid (W_{max}-W_{min}) has taken up energy from the environment at a low temperature in the evaporator by its evaporation (Q_{ev}), while releasing heat in the adsorber at an intermediate temperature level upon adsorption.

The energy required for trajectories I–II and II–III combined is the energy required for desorption (Q_{des}), while the energy released during trajectories III–IV and IV–I is equal to the adsorption energy (Q_{ads}). For practical reasons, in most cases T_1, often called minimum temperature of adsorption T_{ads}, equals T_{con}. The remaining temperatures and pressures used in this cycle cannot be all independently chosen. The condenser and evaporator pressure are inherently linked to their respective temperatures by the vapor–liquid equilibrium of the selected working fluid. For a given working pair, T_2 is fixed by choosing the condenser temperature (and pressure) and related toT_{con} via the maximum

loading isostere (W_{max}). Similarly, T_3 and T_{des} are related through the minimum loading isostere (W_{min}), and T_3 is fixed when the evaporator temperature is selected. In summary, for a given working pair, the operational conditions are fully fixed when evaporator, condenser, and (maximum) desorption temperature are chosen.

Supplementary Note 2.

Thermodynamic calculations methods.

The thermodynamic calculations of adsorption chiller and heat pump cycles are performed by an express method based on the De Lange et al[1]. The coefficient of performance (COP) is adopted to illustrate the energy efficiency of heat pump cycle from a thermodynamic perspective. The COP is defined as the useful energy output divided by the energy required as input. The COP for heating and cooling is written as

$$\mathrm{COP_H} = -\frac{Q_{\mathrm{con}} + Q_{\mathrm{ads}}}{Q_{\mathrm{regen}}} \quad \text{for heat pump} \tag{1}$$

$$\mathrm{COP_C} = \frac{Q_{\mathrm{ev}}}{Q_{\mathrm{regen}}} \quad \text{for cooling} \tag{2}$$

Here, Q_{con} is the energy released during condensation, and Q_{ads} the energy released during the adsorption stage; both have a negative value as energy is rejected from the adsorption system. Q_{ev} is the energy taken up from the environment in the evaporator, and Q_{regen} is the energy required for regeneration of adsorbent, a positive quantity as energy is added to the system. The energy taken up by the evaporator and released by the condenser can be calculated with knowledge of the enthalpy of evaporation, $\Delta_{\mathrm{vap}}H$, by respectively:

$$Q_{\mathrm{ev}} = -\frac{\Delta_{\mathrm{vap}}H(T_{\mathrm{ev}})\rho_{\mathrm{liq}}^{\mathrm{wf}} m_{\mathrm{sorbent}} \Delta W}{M_{\mathrm{w}}} \tag{3}$$

$$Q_{\mathrm{con}} = -\frac{\Delta_{\mathrm{vap}}H(T_{\mathrm{con}})\rho_{\mathrm{liq}}^{\mathrm{wf}} m_{\mathrm{sorbent}} \Delta W}{M_{\mathrm{w}}} \tag{4}$$

Here, $\rho_{\mathrm{liq}}^{\mathrm{wf}}$ and M_{w} is the density and molar mass of the working fluid, and m_{sorbent}

is the amount of adsorbent used in the adsorption cycle. The working capacity, ΔW, is the difference between the maximum load and the minimum load at adsorption and desorption stages ($\Delta W = W_{\max} - W_{\min}$).

Combining the energy required for isosteric heating and isobaric desorption yields the total energy required for regeneration:

$$Q_{\text{regen}} = Q_{\text{I-II}} + Q_{\text{II-III}} \tag{5}$$

The energy gained during the adsorption stage is a combination of the energy gained during isosteric cooling and isobaric adsorption:

$$Q_{\text{ads}} = Q_{\text{III-IV}} + Q_{\text{IV-I}} \tag{6}$$

The energy required for each stage can be described as:

Isosteric heating (Ⅰ-Ⅱ):

$$Q_{\text{I-II}} = \int_{T_{\text{con}}}^{T_2} c_p^{\text{eff}}(T)\text{d}T + \int_{T_{\text{con}}}^{T_2} \rho_{\text{liq}}^{\text{wf}} W_{\max} c_p^{\text{wf}}(T)\text{d}T \tag{7}$$

Isobaric desorption (II−III):

$$Q_{\text{II-III}} = \int_{T_2}^{T_{\text{des}}} c_p^{\text{eff}}(T)\text{d}T + \int_{T_2}^{T_{\text{des}}} \rho_{\text{liq}}^{\text{wf}} \frac{W_{\max} + W_{\min}}{2} c_p^{\text{wf}}(T)\text{d}T - Q_{\text{sorption}} \tag{8}$$

Isosteric cooling (III-IV):

$$Q_{\text{III-IV}} = \int_{T_{\text{des}}}^{T_3} c_p^{\text{eff}}(T)\text{d}T + \int_{T_{\text{des}}}^{T_3} \rho_{\text{liq}}^{\text{wf}} W_{\min} c_p^{\text{wf}}(T)\text{d}T \tag{9}$$

Isobaric adsorption (IV–I):

$$Q_{\text{IV-I}} = \int_{T_3}^{T_{\text{con}}} c_p^{\text{eff}}(T)\text{d}T + \int_{T_3}^{T_{\text{con}}} \rho_{\text{liq}}^{\text{wf}} \frac{W_{\max} + W_{\min}}{2} c_p^{\text{wf}}(T)\text{d}T + Q_{\text{sorption}} \tag{10}$$

Here, c_p^{wf} is the heat capacity of the chosen working fluid and c_p^{eff} is the effective heat capacity of the adsorbent (sorbent) and heat exchanger (hex). The mass of heat exchanger is assumed zero in the efficiency calculations, thus $c_p^{\text{eff}}(T) = c_p^{\text{sorption}}(T)$.

Employing a fixed sorbent heat capacity of 1.0 J·g^{-1}·K^{-1} for all adsorbents will not significantly affect the performance comparison of the different materials under investigation. The energy released during adsorption of the working fluid Q_{sorption} can be written as

$$Q_{\text{sorption}} = \frac{1}{M_{\text{w}}} \int_{W_{\min}}^{W_{\max}} \rho_{\text{liq}}^{\text{wf}} \Delta_{\text{ads}} H(W) \mathrm{d}W \qquad (11)$$

Here, $\Delta_{\text{ads}} H(W)$ is the isosteric enthalpy of adsorption, which can be calculated from adsorption isotherms at two or more different temperatures, using a form of the Clausius–Clapeyron equation:

$$\Delta_{\text{ads}} H_{\text{W}} = R\left(\frac{\partial \ln P}{\partial (1/T)}\right)_W \qquad (12)$$

where $\Delta_{\text{ads}} H_{\text{W}}$, R, P, T, and W represent the isosteric enthalpy of adsorption (kJ·mol^{-1}), universal gas constant (kJ·mol^{-1}·K^{-1}), pressure (kPa), temperature (K), and volume liquid adsorbed (ml$_{\text{water}}$·g$_{\text{sorbent}}^{-1}$), respectively. If the adsorption isotherms of two temperatures are known, equation (12) can be written as

$$\Delta_{\text{ads}} H_W = -\frac{R T_1 T_2}{T_2 - T_1} \ln \frac{P_2}{P_1} \qquad (13)$$

where T_1 and T_2 are the two isotherm temperatures, P_1 and P_2 are pressures at T_1 and T_2, respectively, for given water uptake. In the calculation, the adsorption loading of the isotherms was divided into 10 steps per 0.1 ml·g^{-1} loading. The pressures of three isotherms with the same loading were adopted with linear interpolation. For each step, the natural logarithm of these pressures was plotted depending on reciprocal temperatures of the isotherms, and a linear fit between the points was made.

The concept of the characteristic curve is adopted to transfer the loading from two dependent variables (*P, T*) to one, the adsorption potential, *A*, which equals with opposite sign, to the variation in Gibbs free energy, ΔG

$$A(P,T) = -\Delta G = -RT\ln\frac{P}{P_0(T)} \tag{14}$$

Here P_0 is the vapor pressure of the working fluid. The characteristic curve is then assumed to be temperature-independent and thus the loading at any temperature can be calculated. The amount adsorbed expressed as volume is defined as

$$W = \frac{q(P,T)}{\rho(T)} \tag{15}$$

The equilibrium content between the adsorption potential (*A*) and the amount adsorbed (*W*) can be exclusively determined by the equations (14) and (15). For the isosteric heating stage (I-II), corresponding to the maximum load, and in case of the isosteric cooling stage (III-IV), corresponding to the minimum load. The equations of adsorption potential (*A*) can be respectively written as

$$A(W_{\text{max}}) = -RT_{\text{con}}\ln\frac{P_{\text{ev}}}{P_0(T_{\text{con}})} = -RT_2\,ln\frac{P_{\text{con}}}{P_0(T_2)} \tag{16}$$

$$A(W_{\text{min}}) = -RT_{\text{des}}\ln\frac{P_{\text{con}}}{P_0(T_{\text{des}})} = -RT_3\ln\frac{P_{\text{ev}}}{P_0(T_3)} \tag{17}$$

All the values and equations needed for calculating COP is list in **Supplementary Table 1**

Supplementary Note 3.

Dynamic calculations methods.

The dynamic characteristic of the ADC cycle, namely, the SCP (Specific Cooling Power), is also of primary importance. The SCP depends mainly on the mass and heat transfer in the "adsorber-heat exchanger" unit. In order to evaluate the SCP of EMM-8, kinetics tests were carried out under typical chilling cycle conditions. A simple and commonly used configuration of loose adsorbent grains of diameter Φ= 0.3~0.9 mm, located on a flat metal heat exchanger was studied. The kinetic curves match can be described by the exponential equations,

$$q = \frac{\Delta W(t)}{\Delta W_{t\to\infty}} = 1 - \exp(\frac{-t}{\tau_{\mathrm{ads}}}) \quad \text{for adsorption} \tag{18}$$

$$q = \frac{\Delta W(t)}{\Delta W_{t\to\infty}} = \exp(\frac{-t}{\tau_{\mathrm{des}}}) \quad \text{for desorption} \tag{19}$$

Where q is the dimensionless conversion, and τ_{ads} and τ_{des} are the characteristic times of adsorption and desorption stages.

Indeed, the average SCP obtained in the chilling cycle, restricted by the conversion q= 0.8, can be estimated as

$$\mathrm{SCP}_{0.8} = \frac{\Delta_{\mathrm{vap}} H \cdot \Delta W \cdot 0.8}{M_{\mathrm{w}}(\tau_{0.8\mathrm{ads}} + \tau_{0.8\mathrm{des}})} \tag{20}$$

$$\mathrm{SCP}_{\mathrm{max}} = \frac{\Delta_{\mathrm{vap}} H \cdot \Delta W}{M_{\mathrm{w}}(\tau_{\mathrm{ads}} + \tau_{\mathrm{des}})} \tag{21}$$

where M_w is the water molar weight, $\Delta_{vap}H$ is the enthalpy of evaporation of water, $\tau_{0.8ads}$ and $\tau_{0.8des}$ are the adsorption and desorption times corresponding to the conversion q=0.8.

Base on the water adsorption kinetic curves, the diffusion coefficient of water vapor (moisture diffusivity) within the particles was calculated by using the following equation[2]:

$$q = \frac{\Delta W(t)}{\Delta W_{t\to\infty}} = \frac{12}{D}\sqrt{\frac{D_M t}{\pi}} \qquad (22)$$

where D_M ($cm^2 \cdot s^{-1}$) is the intracrystalline diffusion coefficient, D (mm) is the particle diameter. Plot q versus $t^{\frac{1}{2}}$, and then a straight line with slope $\frac{12}{D}\sqrt{\frac{D_M}{\pi}}$ can be obtained. Thus diffusion coefficient D_M can be calculated from the straight line slope.

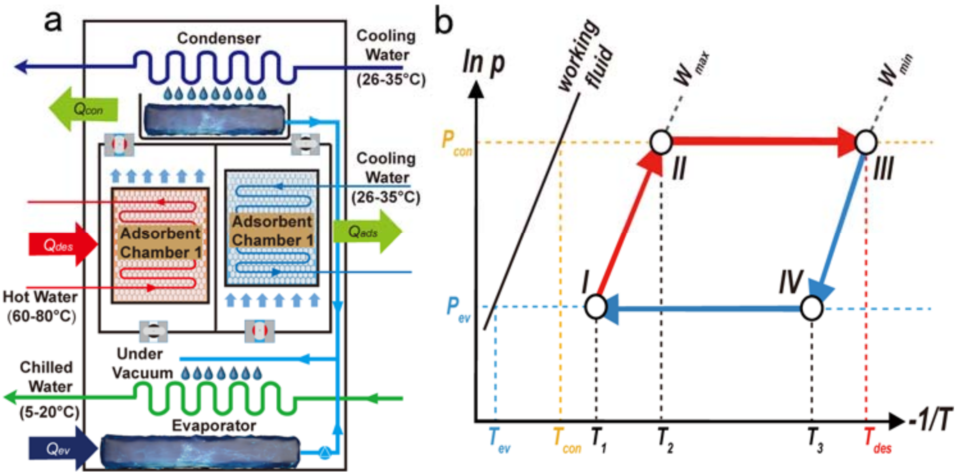

Supplementary Figure 1. Schematic diagram of typical adsorption heat transfer device. a. A diagram of typical adsorptive heat transformation cycle with adsorption stage (Chamber 1) and desorption stage (Chamber 2). Notation: Q_{ev}, Q_{ads}, Q_{des}, and Q_{con} represent evaporation heat, adsorption heat, regeneration heat (or desorption heat) and condensation heat, respectively. The direction of the arrow represents the direction of water flow. **b.** A diagram of typical adsorptive heat transformation cycle plotted in the *P-T* diagram. Notation: T_{ev} and P_{ev} represent the temperature and pressure of the evaporator, T_{con} and P_{con} represent temperature and pressure of the condenser, and T_{des} represent the desorption temperature.

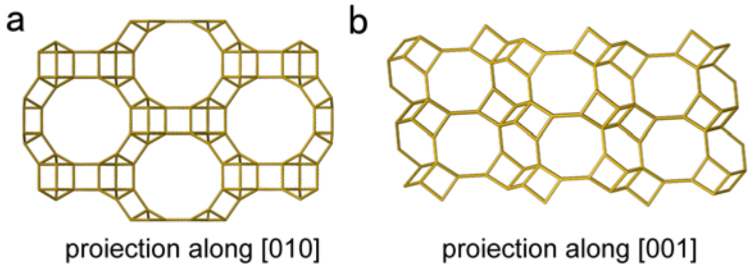

Supplementary Figure 2. Skeleton views of molecular sieves with SFO-topology projected along **a.** [010] and **b.** [001].

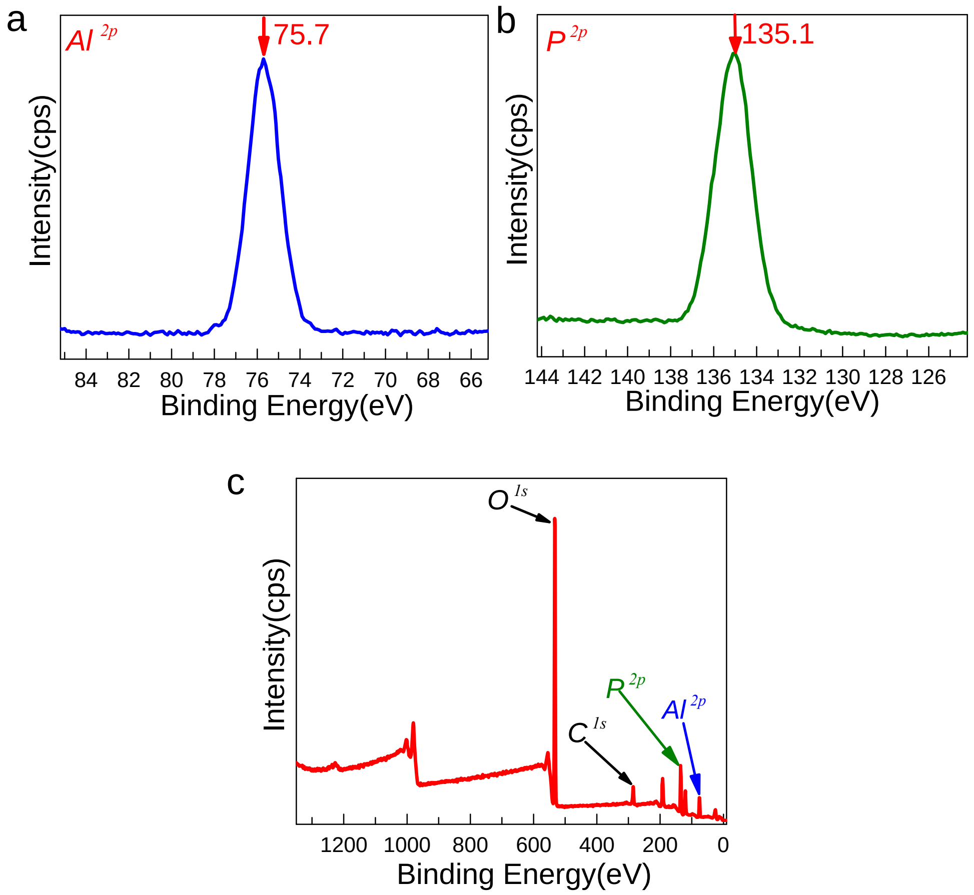

Supplementary Figure 3. XPS spectra of **(a)** *Al* ^{2p}, **(b)** *P* ^{2p}, and **(c)** XPS survey scan of EMM-8.

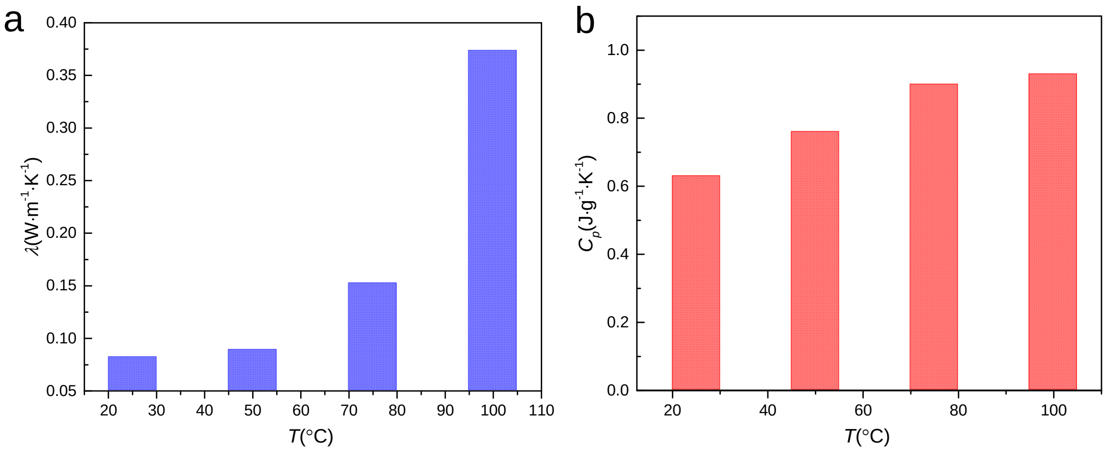

Supplementary Figure 4. a Thermal conductivity, and **b** heat capacity of EMM-8 at 25~100 °C.

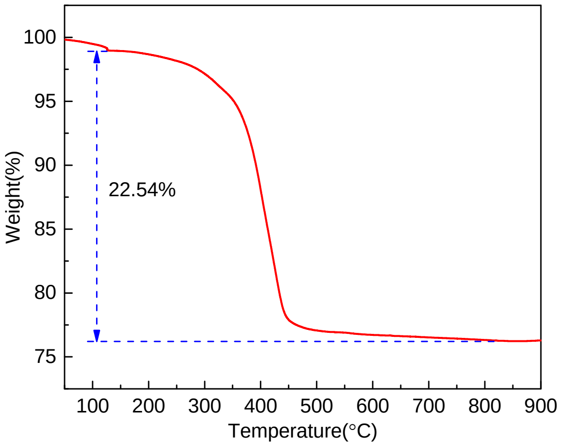

Supplementary Figure 5. Thermogravimetry analysis of as-synthesis EMM-8.

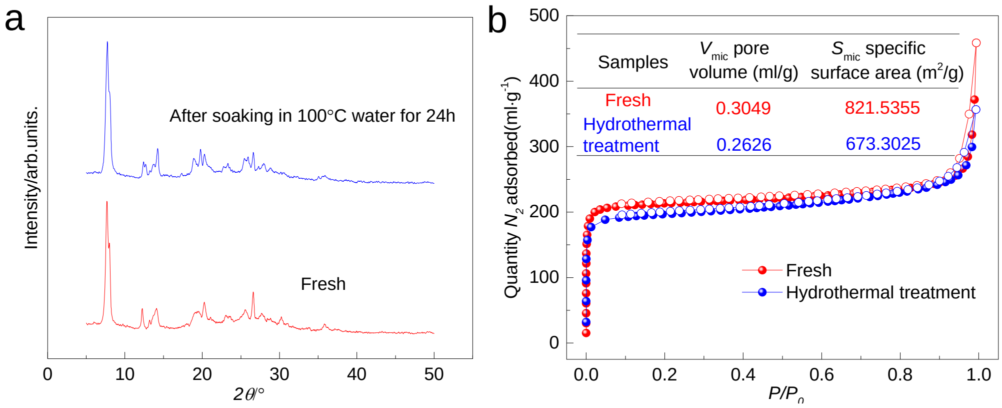

Supplementary Figure 6. The comparisons of typical characterizations between the fresh calcined sample and the hydrothermal treatment sample. a. XRD patterns of the template removed EMM-8 of the one after hydrothermal treatment. **b.** The nitrogen adsorption-desorption isotherms and micropore properties of fresh EMM-8 and that after hydrothermal treatment.

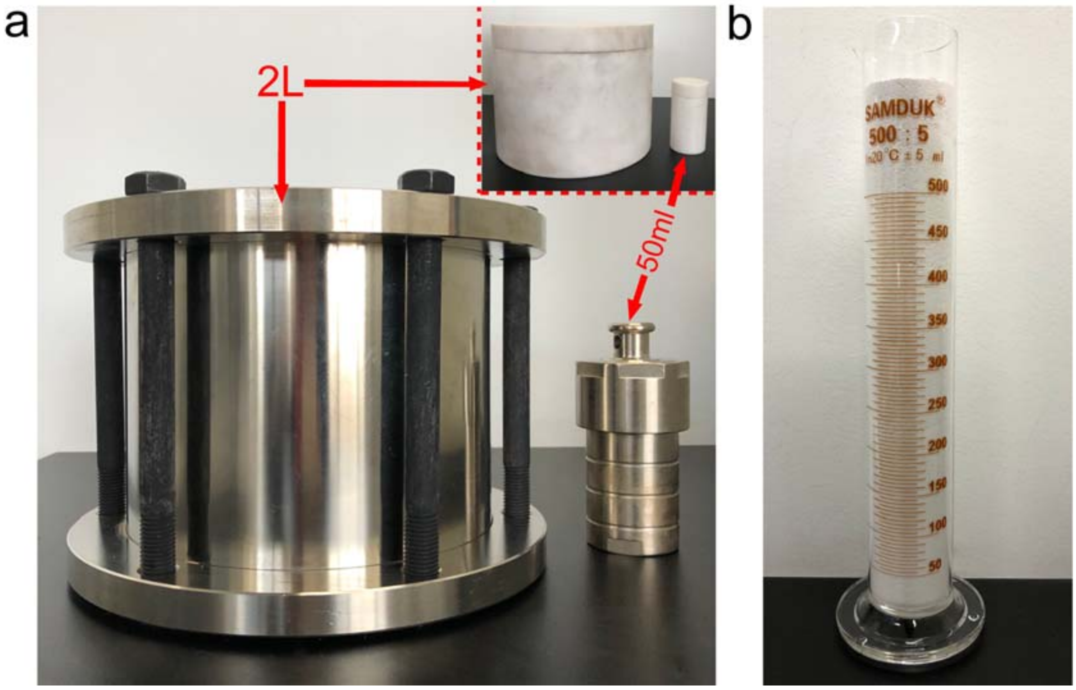

Supplementary Figure 7. Schematic diagrams of scaled-up preparation. a. View of 2L-scale and 50ml-scale Teflon-lined stainless-steel autoclave. **b.** Photo of EMM-8 materials prepared in 2L-scale reactor.

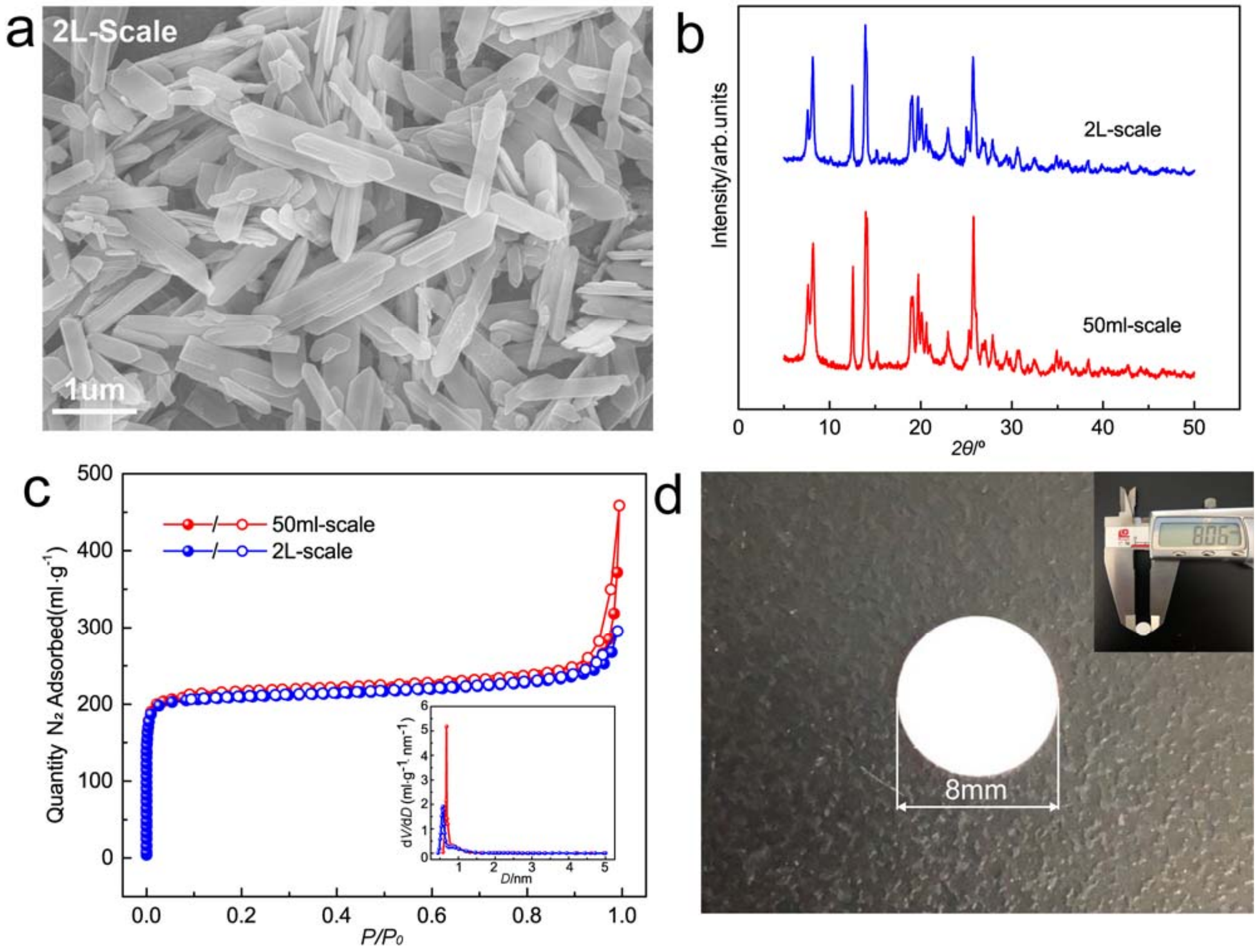

Supplementary Figure 8. Typical characterization of scaled-up samples. a. SEM image of the EMM-8 prepared by 2L-scale Teflon-lined stainless-steel autoclave. **b.** Comparison of XRD patterns between the EMM-8 prepared by 2L-scale and 50ml-scale. **c.** The nitrogen adsorption-desorption isotherms of EMM-8 prepared by 2L-scale and 50ml-scale. The micropore size distribution was shown in the insert map. **d.** Photo of shaped EMM-8.

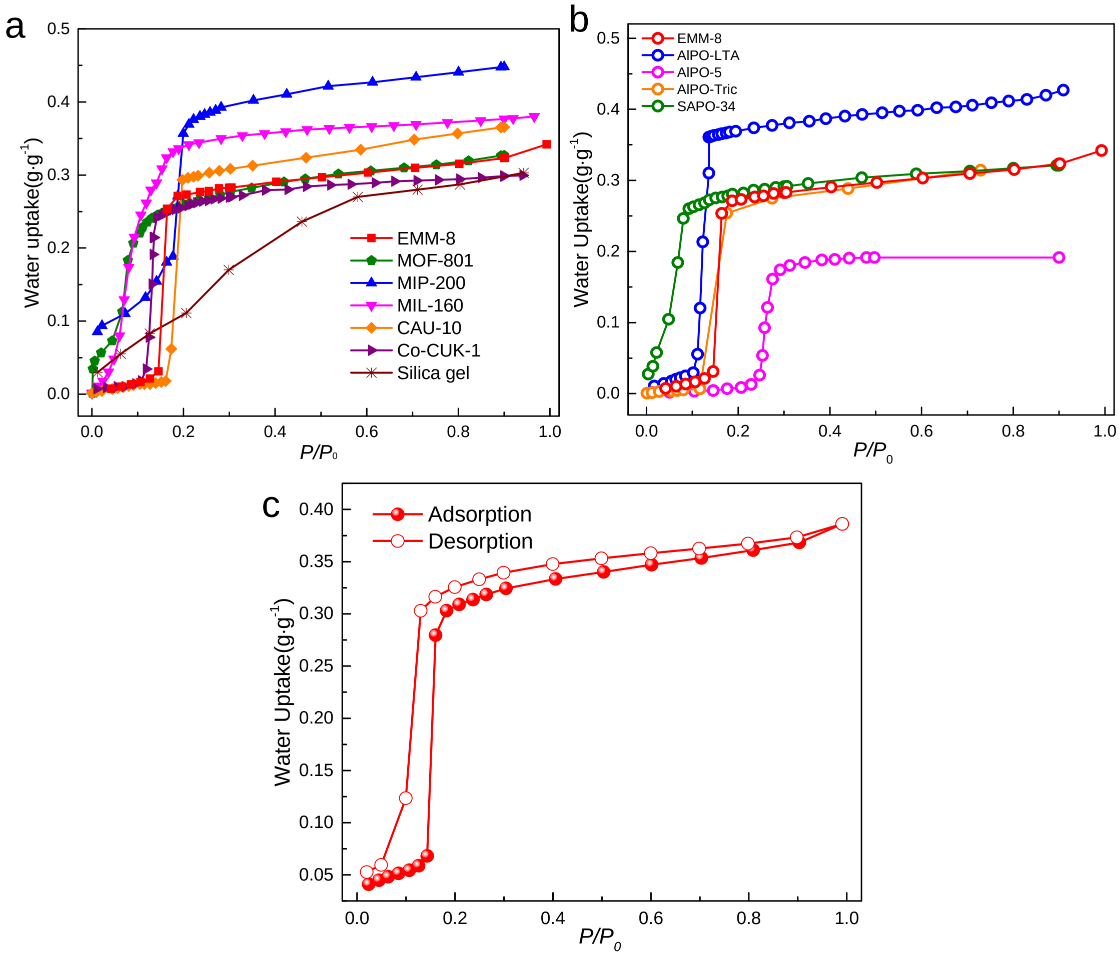

Supplementary Figure 9. Water-adsorption isotherms. a. For EMM-8 (■) and other reference adsorbents: MOF-801 (⬟)[1, 3], MIP-200 (▲)[4], MIL-160 (▼)[5], CAU-10 (◆)[1, 6], Co-CUK-1 (▶)[7] at 30 °C, and silica gel (✳)[8] at 25 °C. **b.** For EMM-8 (○) and other AlPOs & SAPOs adsorbents: AlPO-LTA (○)[9], AlPO-5 (○)[1], AlPO-Tric (○)[10], and SAPO-34 (○)[1] at 30 °C. **c.** Adsorption/desorption isotherms for EMM-8 at 30 °C

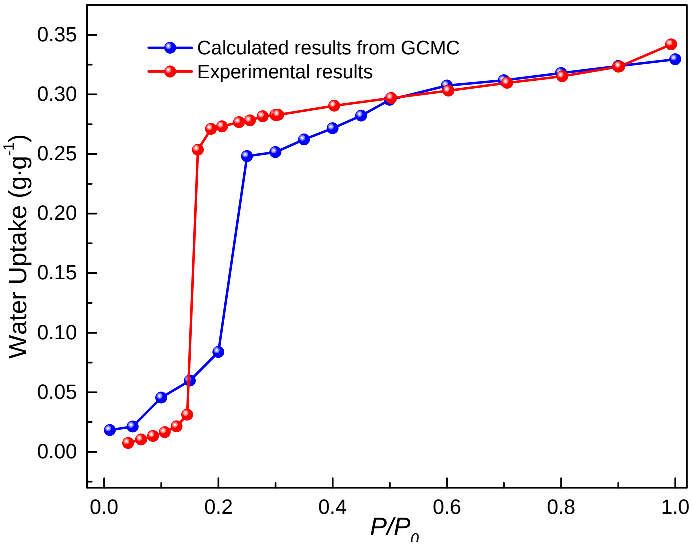

Supplementary Figure 10. Comparison of the GCMC predicted (blue) and experimental (red) water adsorption isotherms for EMM-8 at 30 °C.

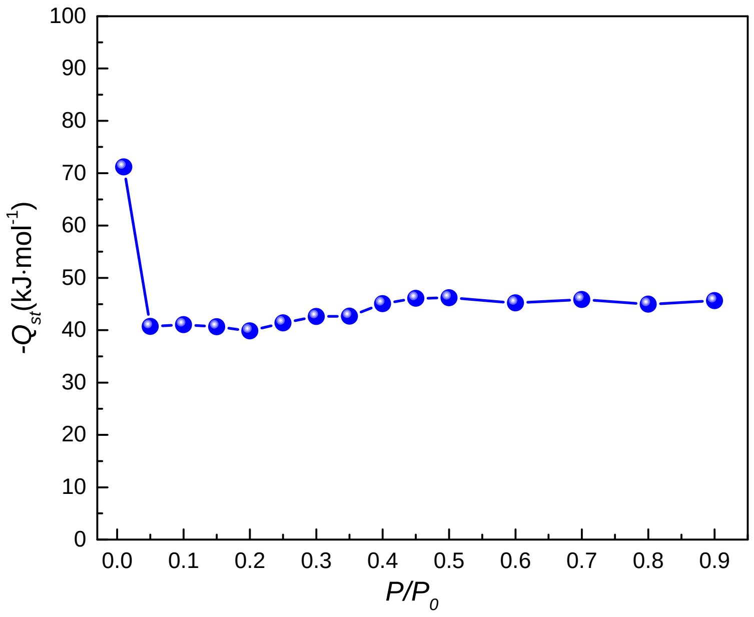

Supplementary Figure 11. GCMC obtained water adsorption enthalpy curve as a function of P/P_0 at 30 °C.

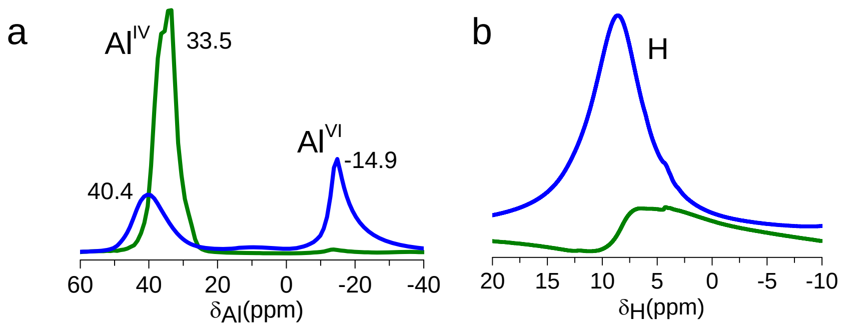

Supplementary Figure 12. a. ^{27}Al and **b.** ^{1}H NMR spectra of completely dried (green) and fully hydrated (blue) EMM-8. Notation: The hydration conditions were 30 °C and P/P_0=0.3.

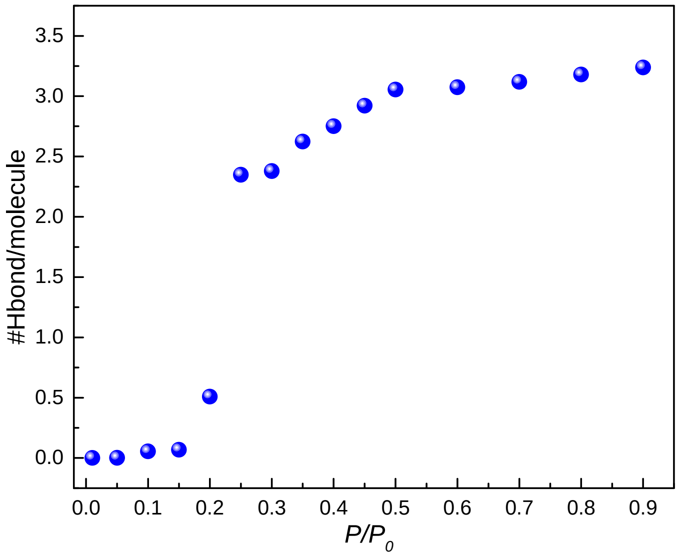

Supplementary Figure 13. Average number of hydrogen-bond (HB) per water molecule as a function of P/P_0.

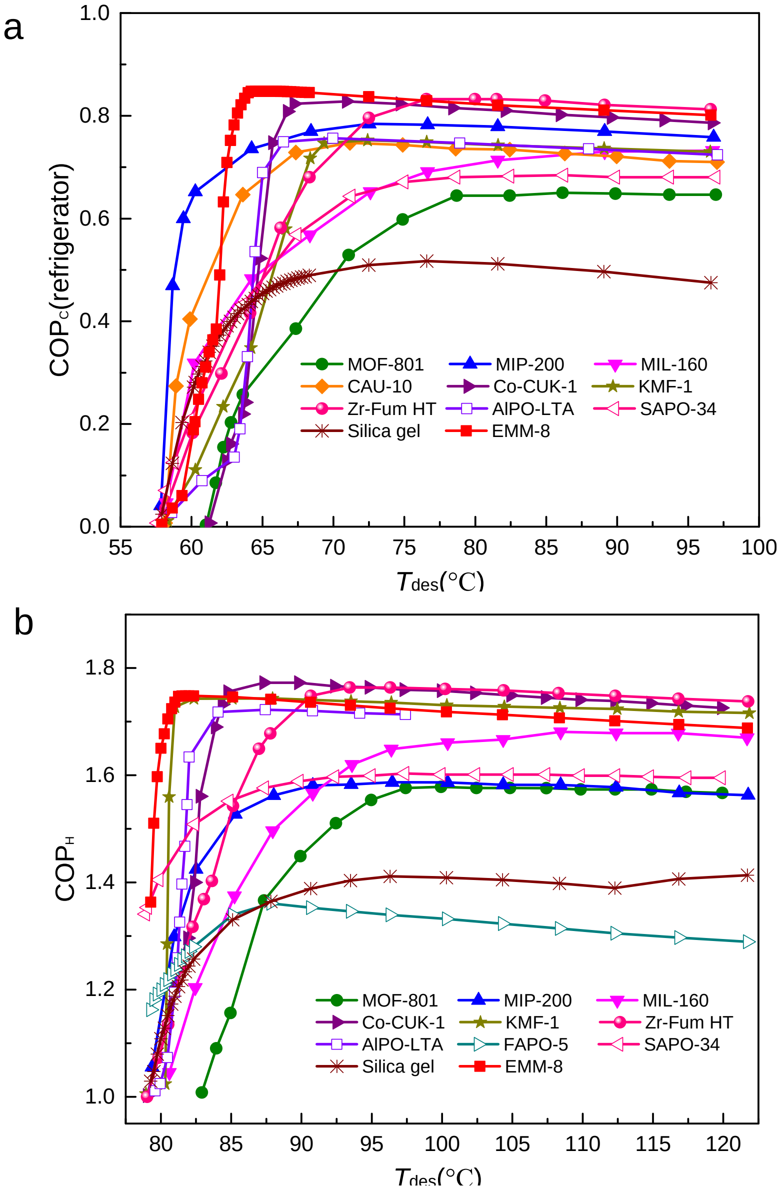

Supplementary Figure 14. Coefficient of performance values for heating and cooling in comparison with reference materials. a. Refrigeration conditions used were T_{ev} = 5 °C and T_{con} = 30 °C. **b.** Heat pump conditions used for calculations were T_{ev} = 15 °C and T_{con} = 45 °C. EMM-8 (■), MOF-801 (⬟)[1, 3], MIP-200 (▲)[4], MIL-160 (▼)[5], CAU-10 (◆)[1, 6], Co-CUK-1 (▶)[7], KMF-1 (★)[11], Zr-Fum HT (●)[12], AlPO-LTA (□)[9], FAPO-5 (▷)[1], SAPO-34 (◁)[1], silica gel (✱)[8].

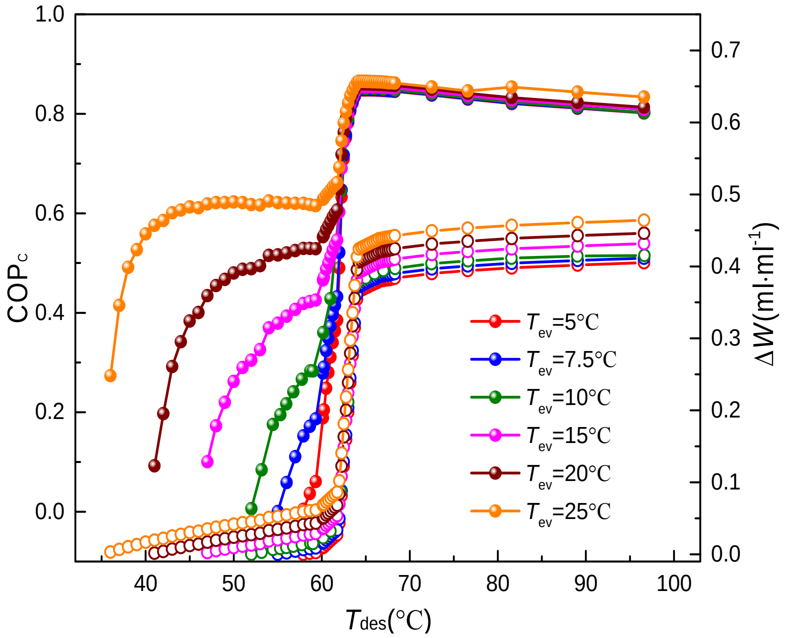

Supplementary Figure 15. Coefficient of Performance (COP_C) plots (Closed symbol) and working capacity based on absorbent volume (Open symbol) of EMM-8 at fixed adsorption temperature (T_{ads} = 30 °C) and condensation temperature (T_{con} = 30 °C) as function of desorption temperatures (T_{des}= 35~105 °C) and evaporation temperatures (T_{ev}=5~25 °C).

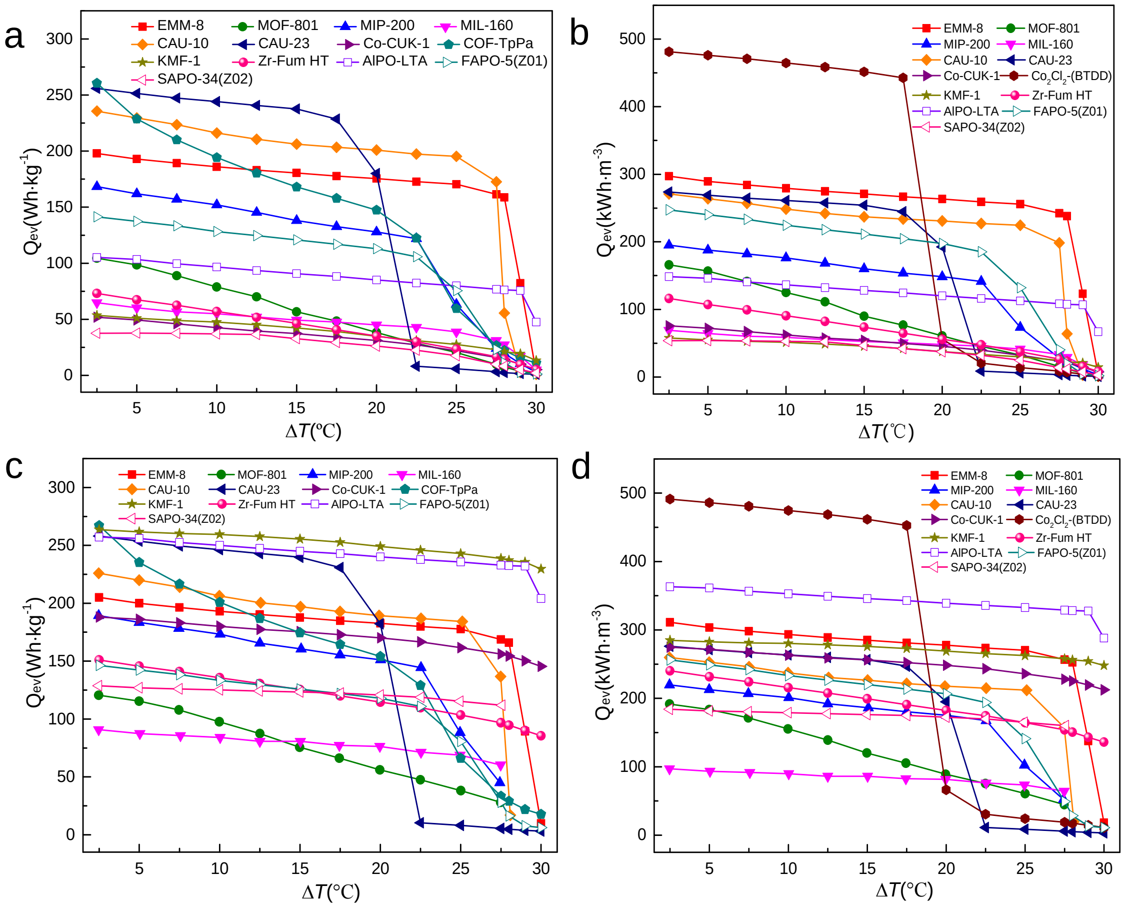

Supplementary Figure 16. Heat transferred from the evaporator in one refrigeration cycle as a function of temperature lift Δ*T* = T_{con} - T_{ev} (T_{con} = 30 °C) at desorption temperature. a, b. T_{des} = 65 °C (**a.** gravimetric heat transferred, **b.** volumetric heat transferred), and **c, d.** T_{des} = 70 °C (**c.** gravimetric heat transferred, **d.** volumetric heat transferred). EMM-8 (■), MOF-801 (⬟)[1, 3], MIP-200 (▲)[4], MIL-160 (▼)[5], CAU-10 (◆)[1, 6], CAU-23 (◀)[13], Co-CUK-1 (▶)[7], Co_2Cl_2-(BTDD) (⬣)[14], COF-TpPa (⬟)[15], KMF-1 (★)[11], Zr-Fum HT (●)[12], AlPO-LTA (□)[9], FAPO-5 (▷)[1], SAPO-34 (◁)[1].

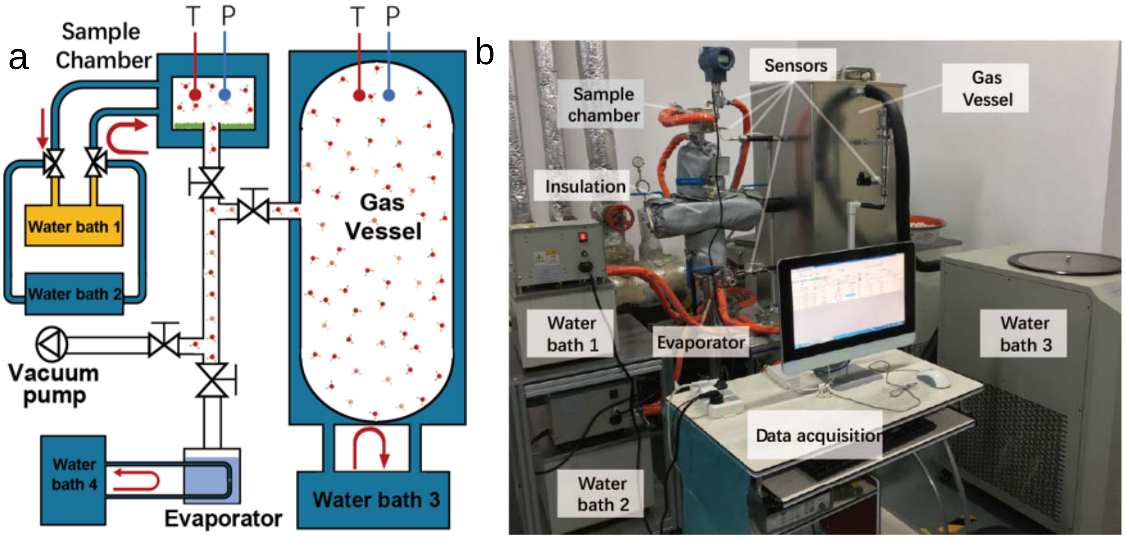

Supplementary Figure 17. View and scheme of self-constructed apparatus for the test of water adsorption kinetics installed at Research Center of Solar Power & Refrigeration labs in Shanghai Jiao Tong University.

a b

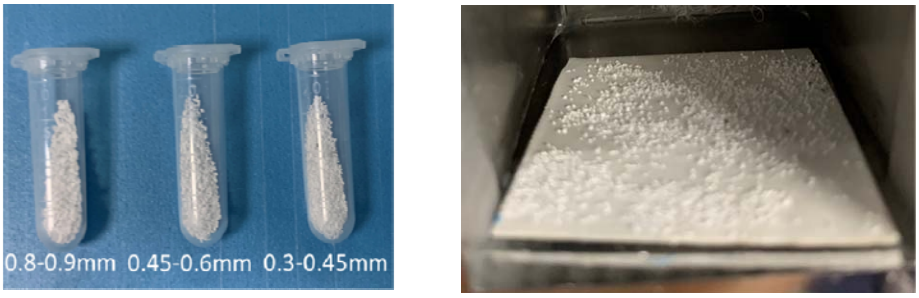

Supplementary Figure 18. a. The template-removed EMM-8 grains with different sizes and **b.** the view of the tested sorbents.

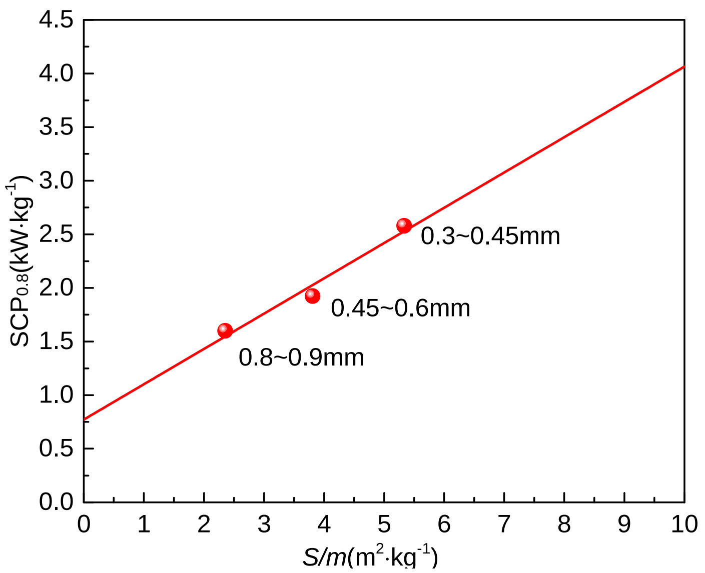

Supplementary Figure 19. $SCP_{80\%}$ as function of the *S/m* ratio for the flat adsorbent beds, composed of the grains of diameter dimension = 0.3~0.45 mm, 0.45~0.6 mm, 0.8~0.9 mm. The operating conditions were T_{ev}=10 °C, T_{con}=30 °C, and T_{des}=80 °C. $SCP_{80\%}$ refers to the mass specific cooling power of the conversion q= 0.8, $kW\cdot kg_{ads}^{-1}$.

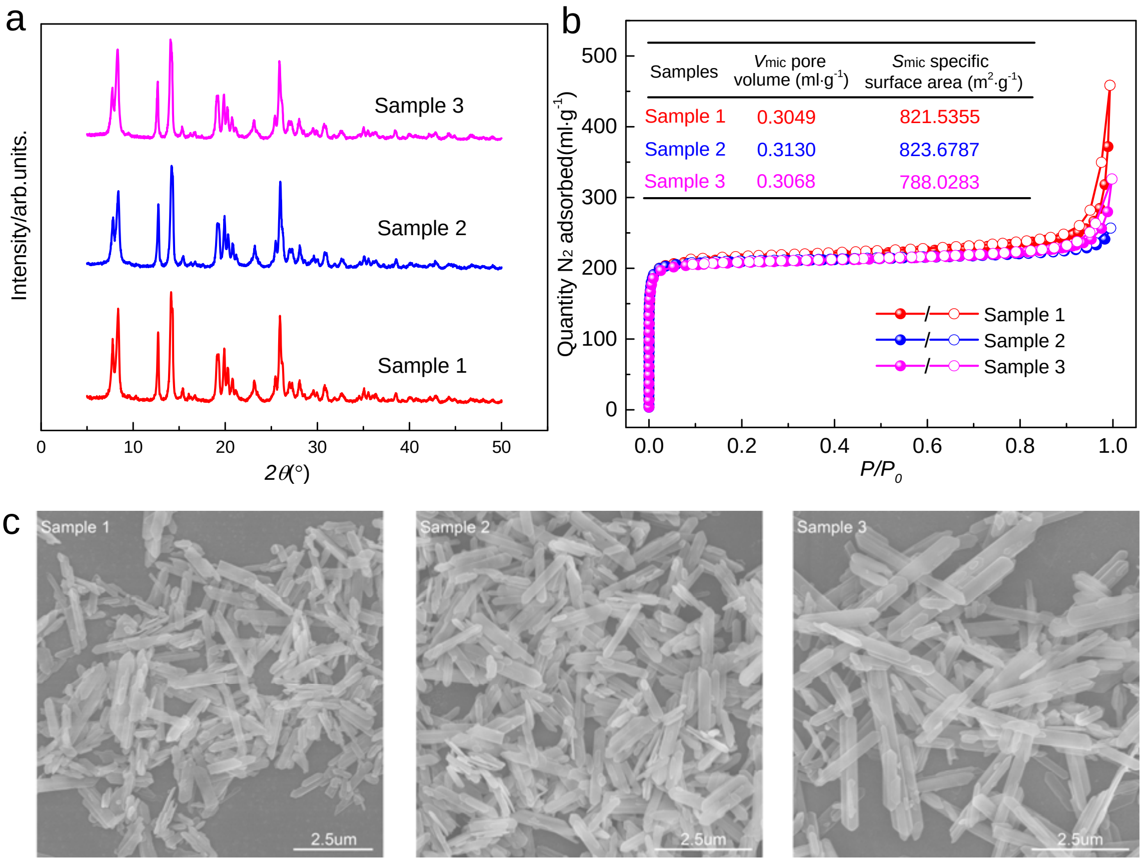

Supplementary Figure 20. Repeatability of the synthesis of EMM-8. a. XRD patterns of samples 1-3 obtained by repetitive fabrication progressesthree times. **b.** Nitrogen sorption isotherms of samples 1-3 taken at 77 K, the inset image gives the pore size distribution of the samples. c. SEM images of samples 1-3.

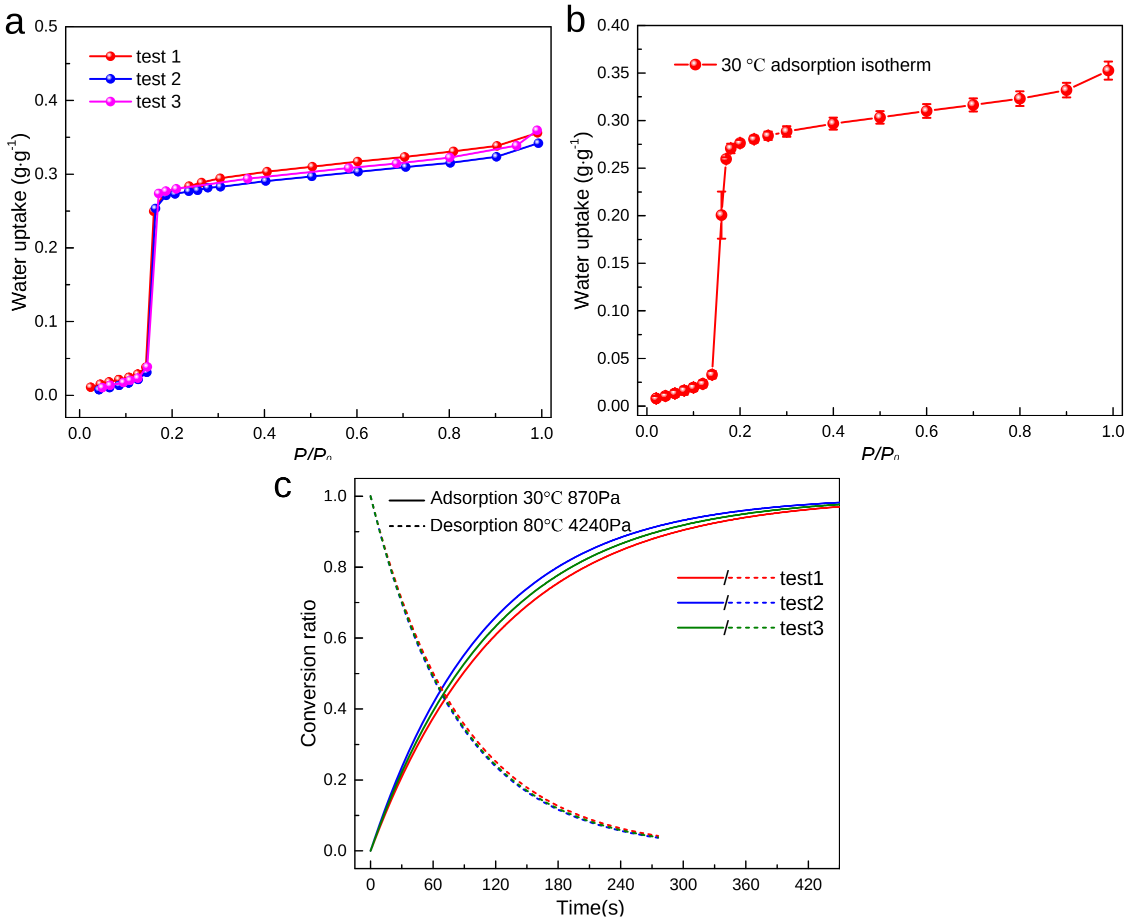

Supplementary Figure 21. Repeatability and uncertainty analysis of water adsorption test. a. Water adsorption isotherm of three tests at 30 ℃. **b.** Water uptake with error bars for EMM-8 at 30 ℃. **c.** Dimensionless water uptake curves during adsorption (30 ℃ and 870 Pa) and desorption (80 ℃ and 4200 Pa) of three tests. The size of EMM-8 grain is 0.45~0.6 mm.

Uncertainty analysis.

The uncertainty analysis is conducted based on the standard error analysis method. Water sorption isotherms were measured by a 3H-2000 PW intelligent gravimetric analyzer (IGA, Beishide Instrument Technology Co., Ltd.). The electronic balance of intelligent gravimetric analyzer is customized, and its mass inaccuracy is ±0.001 mg.

The inaccuracies of pressure sensor (INFICON, CDG025D) and temperature sensor (OMEGA PT100) are ±0.1Pa and ±0.1℃, respectively. A confidence level of the all uncertainties is 95%. Based on Equation (23)-(28), the uncertainty analysis of water uptake q(g·g^{-1}) at 30 ℃ was carried out. Where, m_i, m_0, q_i, and $\bar{q}$ represent the real-time quality of EMM-8 with the adsorption process, the quality of dehydrated EMM-8, the water uptake for multiple measurement, and the average water uptake of multiple measurement, respectively. μ and E represent the uncertainty and relative uncertainty. The uncertainty of water uptake is calculated in the range of 0.22~1.80 % at relative pressure $P/P_0 \leq 0.16$,which corresponds to the main region of interest. For example, the uncertainties of water uptake q(g·g^{-1}) at P/P_0 = 0.02 and P/P_0 = 0.16 are 1.80 % and 0.22 %, respectively. However, this value drops to less than 0.1% at higher relative pressure $P/P_0 > 0.16$.

$$\mu_{q,\mathrm{A}} = t\sqrt{\frac{\sum_{i=1}^{n}(q_\mathrm{i}(P,T)-\bar{q})^2}{\mathrm{n}(\mathrm{n}-1)}} \tag{23}$$

$$q(P,T) = \frac{m_\mathrm{i}-m_0}{m_0} \tag{24}$$

$$\ln q(P,T) = \ln(m_\mathrm{i}-m_0) - \ln m_0 \tag{25}$$

$$E_{q,\mathrm{B}} = \sqrt{\left(\frac{\partial \ln q(P,T)}{\partial m_0}\mu_{m_0}\right)^2 + \left(\frac{\partial \ln q(P,T)}{\partial m_\mathrm{i}}\mu_m\right)^2} = \sqrt{\left(-\frac{m_\mathrm{i}\mu_{m_0}}{m_0(m_\mathrm{i}-m_0)}\right)^2 + \left(\frac{\mu_m}{m_\mathrm{i}-m_0}\right)^2} \tag{26}$$

$$\mu_{q,\mathrm{B}} = E_{q,\mathrm{B}} \times q(P,T) \tag{27}$$

$$\mu_q = \sqrt{{\mu_{q,\mathrm{A}}}^2 + {\mu_{q,\mathrm{B}}}^2} \tag{28}$$

Supplementary Table 1. All the values and equations needed for calculating COP

Steps	Purpose	Values needed for calculate	Equations needed for calculating	Remarks
1	Characteristic curve (A-W)	$q(P,T)$, P, T	$A(P,T) = -RT\ln\frac{P}{P_0(T)}$<br>$W = \frac{q(P,T)}{\rho(T)}$	(A-W) is assumed to be temperature-independent
2	Adsorption enthalpy curve (W-$\Delta_{ads}H_w$)	T_1, T_2, T_3 & P_1, P_2, P_3 (W=const)	$\Delta_{ads}H_w = R(\frac{\partial \ln P}{\partial(1/T)})_w$	Depend on sorption isotherms at different T
3	Maximum and minimum load (W_{max} & W_{min})	T_{ev}, T_{con}, T_{des}, P_{ev}, P_{con}, P_{des}	$A(W_{max}) = -RT_{con}\ln\frac{P_{ev}}{P_0(T_{con})}$<br>$A(W_{min}) = -RT_{des}\ln\frac{P_{con}}{P_0(T_{des})}$<br>Characteristic curve（$A$-$W$）	Depend on characteristic curve and adsorption potential at W_{max}/W_{min}

Continued Supplementary Table 1.

Steps	Purpose	Values needed for calculate	Equations needed for calculating	Remarks
4	T_2, T_3	T_{ev}, T_{con}, T_{des},<br>P_{ev}, P_{con}, P_{des}	$A(W_{max}) = -RT_{con}\ln\frac{P_{ev}}{P_0(T_{con})} = -RT_2\ln\frac{P_{con}}{P_0(T_2)}$<br>$A(W_{min}) = -RT_{des}\ln\frac{P_{con}}{P_0(T_{des})} = -RT_3\ln\frac{P_{ev}}{P_0(T_3)}$	Point I and II are linked by W_{max}, point III and IV are linked by W_{min}
5	$Q_{sorption}$	W_{max}, W_{min}, ρ_{liq}^{wf}, M_w<br>Adsorption enthalpy curve<br>(W-$\Delta_{ads}H_w$)	$Q_{sorption} = \frac{1}{M_w}\int_{W_{min}}^{W_{max}}\rho_{liq}^{wf}\Delta_{ads}H(W)\mathrm{d}W$	Adsorption enthalpy integral between W_{max} and W_{min}
6	Q_{ev}, Q_{con}	$\Delta_{vap}H(T_{ev})$, $\Delta_{vap}H(T_{con})$,<br>$m_{sorption}$, ΔW	$Q_{ev} = -\frac{\Delta_{vap}H(T_{ev})\rho_{liq}^{wf}m_{sorbent}\Delta W}{M_w}$<br>$Q_{con} = \frac{\Delta_{vap}H(T_{con})\rho_{liq}^{wf}m_{sorbent}\Delta W}{M_w}$<br>$\Delta W = W_{max} - W_{min}$	Q_{ev} is positive,<br>Q_{con} is negative

Continued Supplementary Table 1.

Steps	Purpose	Values needed for calculate	Equations needed for calculating	Remarks
7	The energy for each stage $Q_{\text{I-II}}$, $Q_{\text{II-III}}$, $Q_{\text{III-IV}}$, $Q_{\text{IV-I}}$	T_{con}, T_2, T_3, T_{des}, $W_{\max}$, $W_{\min}$, $\rho_{\text{liq}}^{\text{wf}} c_p^{\text{wf}}(T)$, Q_{sorption}	$Q_{\text{I-II}} = \int_{T_{\text{con}}}^{T_2} c_p^{\text{eff}}(T)\text{d}T + \int_{T_{\text{con}}}^{T_2} \rho_{\text{liq}}^{\text{wf}} W_{\max} c_p^{\text{wf}}(T)\text{d}T$ $Q_{\text{II-III}} = \int_{T_2}^{T_{\text{des}}} c_p^{\text{eff}}(T)\text{d}T + \int_{T_2}^{T_{\text{des}}} \rho_{\text{liq}}^{\text{wf}} \frac{W_{\max}+W_{\min}}{2} c_p^{\text{wf}}(T)\text{d}T - Q_{\text{sorption}}$ $Q_{\text{III-IV}} = \int_{T_{\text{des}}}^{T_3} c_p^{\text{eff}}(T)\text{d}T + \int_{T_{\text{des}}}^{T_3} \rho_{\text{liq}}^{\text{wf}} W_{\min} c_p^{\text{wf}}(T)\text{d}T$ $Q_{\text{IV-I}} = \int_{T_3}^{T_{\text{con}}} c_p^{\text{eff}}(T)\text{d}T + \int_{T_3}^{T_{\text{con}}} \rho_{\text{liq}}^{\text{wf}} \frac{W_{\max}+W_{\min}}{2} c_p^{\text{wf}}(T)\text{d}T + Q_{\text{sorption}}$ $c_p^{\text{eff}} \approx c_p^{\text{sorption}} \approx 1.0 J \cdot g^{-1} \cdot K^{-1}$	$Q_{\text{I-II}}$, $Q_{\text{II-III}}$ is positive, $Q_{\text{III-IV}}$, $Q_{\text{IV-I}}$ is negative
8	Q_{regen}, Q_{ads}	$Q_{\text{I-II}}$, $Q_{\text{II-III}}$, $Q_{\text{III-IV}}$, $Q_{\text{IV-I}}$,	$Q_{\text{regen}} = Q_{\text{I-II}} + Q_{\text{II-III}}$ $Q_{\text{ads}} = Q_{\text{III-IV}} + Q_{\text{IV-I}}$	Q_{regen} is positive, Q_{ads} is negative
9	COP	Q_{ev}, Q_{con}, Q_{regen}, Q_{ads}	$\text{COP}_{\text{H}} = -\frac{Q_{\text{con}} + Q_{\text{ads}}}{Q_{\text{regen}}}$ $\text{COP}_{\text{C}} = \frac{Q_{\text{ev}}}{Q_{\text{regen}}}$	$1 \le \text{COP}_{\text{H}} \le 2$ $\text{COP}_{\text{C}} \le 1$

Supplementary Table 2. Test data of pore volume and specific surface area of EMM-8 obtained in 2L-scale and 50ml-scale reactor.

Samples	Pore volume (ml/g)		Specific surface area (m^2/g)	
	V_{total}	V_{mic}	S_{total}	S_{mic}
50ml-scale	0.5929	0.3049	879.6338	821.5355
2L-scale	0.4563	0.3017	842.2128	790.3089

Supplementary Table 3. The results of Al / P atomic ratio were obtained by TEM-EDS, ICP-OES, and XPS.

Sample	Al / P atomic ratio		
	TEM-EDS	ICP-OES	XPS
EMM-8	0.959	1.033	1.022

Supplementary Table 4. Coefficient of performance of EMM-8 and reference water adsorbents.

Materials	COP for cooling (T_{ev} = 5 ℃, T_{con} = 30 ℃)				COP for heating (T_{ev} = 15 ℃, T_{con} =45 ℃)			ref
	$COP_{c,max}$ (T_{des},℃)	different T_{des} (℃)			$COP_{H,max}$ (T_{des},℃)	different T_{des} (℃)		
		65	70	80		80	100	
EMM-8	0.85 (65)	0.85	0.84	0.82	1.75 (82)	1.65	1.72	This work
MOF-801	0.65 (86)	0.30	0.49	0.64	1.58 (100)	/	1.58	1
MIP-200	0.78 (70)	0.74	0.78	0.78	1.59 (96)	1.15	1.59	4
MIL-160	0.73 (97)	0.50	0.60	0.71	1.68 (108)	/	1.66	5
CAU-10	0.75 (71)	0.68	0.74	0.735	1.28 (102)	/	1.18	1,6
CAU-23	0.41 (82)	0.32	0.37	0.40	1.30 (117)	1.04	1.28	13
Co-CUK-1	0.83 (71)	0.57	0.83	0.81	1.77 (87)	1.11	1.76	7
Co_2Cl_2-(BTDD)	0.75 (97)	0.54	0.61	0.70	1.64 (122)	1.14	1.59	14
COF-TpPa	0.73 (72.5)	0.72	0.73	0.72	1.55 (104)	1.11	1.54	15
KMF-1	0.75 (70)	0.42	0.75	0.74	1.74 (85)	1.02	1.73	11
Zr-Fum HT	0.83 (80)	0.48	0.73	0.83	1.76 (93.5)	1.09	1.76	12
AlPO-LTA	0.76 (70)	0.69	0.76	0.75	1.72 (87)	1.03	1.71	9
AQSOA-Z01	0.70 (62)	0.69	0.685	0.66	1.36 (88)	1.20	1.33	1
AQSOA-Z02	0.68 (86)	0.48	0.62	0.68	1.61 (105~107)	1.41	1.61	1
AQSOA-Z05	0.11 (89)	0.086	0.109	0.109	1.01 (108)	/	1.005	1
Silica gel	0.52 (77)	0.45	0.50	0.51	1.41 (96)	1.11	1.41	8

Supplementary Table 5. Water sorption properties and specific energy capacity of EMM-8 and reference water adsorbents.

Material	Crystal density	V[a] (cm^3 g^{-1})		Porosity	$\langle -\Delta_{ads}H \rangle$ ($kJ \cdot mol^{-1}$)	Working capacity[d, f]		Specific energy capacity[e,f]		Ref
	($g\ cm^{-3}$)	V_{mic}	V_{total}			($g \cdot g^{-1}$)	($ml \cdot ml^{-1}$)	($Wh \cdot kg^{-1}$)	($kWh \cdot m^{-3}$)	
EMM-8	1.50	0.30	0.59	0.45	46.76	0.256	0.384	176	263	This work
MOF-801	1.59	0.45	/	0.72	58.40	0.056	0.089	38	61	1,3
MIP-200	1.16	0.40[b]	/	0.46	51.20[f]	0.187	0.217	128	148	4
MIL-160	1.07	0.398	/	0.43	50.64[f]	0.066	0.071	45	48	5
CAU-10	1.15	0.27	/	0.31	53.50	0.293	0.337	201	231	1, 6
CAU-23	1.07	0.48	/	0.51	48.20	0.262	0.280	180	193	13
Co-CUK-1	1.46	0.26[c]	/	0.38	48.65[f]	0.045	0.066	31	45	7
Co_2Cl_2-(BTDD)	0.69	/		/	46.43[f]	0.119	0.082	81	56	14
COF-TpPa	/	/		/	45.00	0.215	/	147	/	15
KMF-1	1.08	/	0.473	/	52~57	0.051	0.055	35	38	11
Zr-Fum HT	1.59	0.389	0.553	0.62	45~52	0.051	0.081	35	56	12
AlPO-LTA	1.41	0.32	/	0.45	55.58[f]	0.124	0.175	85	120	9, 16
FAPO-5	1.75	0.15	/	0.26	56.10	0.165	0.289	113	198	1, 17
SAPO-34	1.43	0.212	/	0.30	57.00	0.038	0.054	26	37	1, 18
AlPO-5	1.75	/	/	/	52.6	0.135	0.236	92	162	1
Silica gel	0.72	0.38	0.40	0.27	54.64[f]	0.076	0.055	52	37	8

[a] Based on N_2 sorption isotherms at 77 K.

[b] After a simple washing in boiling water at room temperature.

[c] Based on CO_2 sorption isotherms at 194.5 K.

[d] Working capacity deduced from one refrigeration cycle at T_{ev}= 10 °C, T_{con}= 30 °C, and T_{des}=65 °C;

[e] Heat transferred from the evaporator in one refrigeration cycle at T_{ev}= 10 °C, T_{con}= 30 °C and T_{des}= 65 °C;

[f] Values for MOF-801, MIP-200, MIL-160, CAU-10, CAU-23, Co-CUK-1, Co-CUK-1, Ni-CUK-1, Mg-CUK-1, Co_2Cl_2-(BTDD), COF-TpPa, KMF-1, Zr-Fum HT, AlPO-LTA, FAPO-5, SAPO-34, AlPO-5, Silica gel were calculated by characteristic curves and water adsorption data taken from references.

Supplementary Table 6. Main features of the tested dynamics adsorber

Sorbent grain size, mm	0.3~0.45/0.45~0.6/0.8~0.9
HT area/dry mass (*S/m*), m^2 /kg	5.33/3.81/2.53

Supplementary Table 7. Operating conditions of dynamics tests.

Evaporation temperature, T_{ev} [°C]	5/10/15
Condensation temperature, T_{con} [°C]	30
Regeneration temperature, T_{des} [°C]	65/70/80

Supplementary Table 8. Diffusion coefficient (D_M~$10^{-7}cm^2·s^{-1}$) of water vapor on EMM-8

Cycle	Operating condition			Slope	$\frac{12}{D\sqrt{\pi}}$	D_M
	T (℃)	P (Pa)	Diameter D (mm)	($s^{-1/2}$)	(mm^{-1})	($10^{-7}cm^2·s^{-1}$)
1	30	1200	0.3~0.45	0.0985	18.0528	2.98
2	30	1200	0.45~0.6	0.0787	12.8950	3.72
3	30	1200	0.8~0.9	0.0686	7.9646	7.42
4	30	870	0.45~0.6	0.0628	12.8950	2.37
5	30	1700	0.45~0.6	0.1154	12.8950	8.01

Supplementary Table 9. Specific cooling power (SCP) values and sorption characteristic times of shaped EMM-8 obtained from kinetic water sorption measurements under different sizes. The operating condition of tests was $T_{ev}/T_{con}/T_{des}$ = 10/30/80℃.

Cycle	Size	Kinetic evaluation results				
	Diameter (mm)	τ (s)	$\tau_{0.8}$ (s)	ΔW_∞ (g g^{-1})	$SCP_{80\%}$ (kW kg^{-1})	SCP_{max} (kW kg^{-1})
1	0.3~0.45	129.444	208.332	0.272	2.579	5.189
2	0.45~0.6	173.544	279.308	0.272	1.923	3.868
3	0.8~0.9	208.608	335.742	0.272	1.600	3.220

Supplementary Table 10. Specific cooling power (SCP) values and sorption characteristic times of shaped EMM-8 obtained from kinetic water sorption measurements under several operating conditions. The size of EMM-8 grain is 0.45~0.6mm.

Cycle	Operating temp. (℃)			Kinetic evaluation results				
	T_{ev}	T_{con}	T_{des}	τ (s)	$\tau_{0.8}$ (s)	ΔW_{∞} (g g^{-1})	$SCP_{80\%}$ (kW kg^{-1})	SCP_{max} (kW kg^{-1})
1	5	30	65	313.935	505.259	0.246	1.104	2.221
2	5	30	70	277.852	447.185	0.257	1.304	2.623
3	10	30	65	276.597	445.166	0.254	1.127	2.268
4	10	30	70	240.514	387.092	0.265	1.353	2.722
5	10	30	80	173.544	279.308	0.272	1.923	3.868
6	15	30	65	229.401	369.207	0.263	1.402	2.821
7	15	30	70	193.318	311.133	0.274	1.733	3.487

Supplementary Table 11. Specific cooling power (SCP) values comparison for different materials under typical cooling conditions.

Material	T_{ev} (℃)	T_{con} (℃)	T_{des} (℃)	S/m ($m^2\ kg^{-1}$)	Size (mm)	ΔW ($g\ g^{-1}$)	$SCP_{80\%}$[a] ($kW\ kg^{-1}$)	Ref
Materials Grain								
NH2-MIL-125	10	30	75	3.5	0.4~0.5	0.39	1.1	19, 20
MOF-801	5	30	85	4.4	0.4~0.5	0.22	1.6	21, 22
KMF-1	10	35	70	1.575	0.5~1.5	0.30	0.55	11
SAPO-34	15	28	90	3.62	0.6~0.7	/	0.498[b]	23
EMM-8	10	30	70	3.81	0.45~0.60	0.27	1.353	This work
Materials Coating on Full Heat Exchanger								
CAU-10(Al)-H[d]	10	30	70	/	0.55	/	1.204[c]	24
CAU-10(Al)-H[d]	14	34	70	/	0.55	/	1.369[c]	24
Al fumarate (Basolite A520)[d]	18	30	90	/	0.3~0.33	/	1.394[c]	25
SAPO-34	15	28	90	11.19	0.1	/	0.675[b]	23

[a] Mass specific cooling power of the conversion q= 0.8, $kW\ kg_{ads}^{-1}$.

[b] Cycle time= 5 min.

[c] Mass specific cooling power of the conversion q= 0.9, $kW\ kg_{ads}^{-1}$.

[d] Coating with binder.

Supplementary Table 12. Comparison of raw material's cost. All prices refer to Macklin's official website (www.macklin.cn accessed 5 July 2021).

Materials	Raw materials	Cost ($/kg$_{raw\ materials}$)	Raw materials consumption (Kg/kg$_{Sorbent}$)	Total cost ($/kg$_{sorbent}$)	Ref
EMM-8	H_3PO_4	5.05	0.73	122.74	This work
	Pseudo-boehmite	13.33	1.27		
	4-DMAPy	74.59	1.36		
	HF	4.85	0.14		
MOF-801	Fumaric acid	12.11	0.71	380.24	26
	$ZrOCl_2$ • $8H_2O$	187.70	1.98		
MIP-200	H4mdip	492174.13	0.41	201855.57	4
	$ZrCl_4$	78.26	0.82		
MIL-160	2,5-furandicarboxylic acid	1329.79	0.65	879.77	5
	$AlCl_3$ • $6H_2O$	13.94	1.01		
	NaOH	7.83	0.17		
CAU-23	H_2TDC	307.53	0.96	331.17	13
	NaOH	7.83	0.44		
	$AlCl_3$	22.99	0.56		
	$NaAlO_2$	178.41	0.11		

Co-CUK-1	2,4-pdcH_2	1769.99	0.62	1787.74	27
	KOH	16.08	41.15		
	$CoCl_2 \cdot 6H_2O$	44.08	0.65		
Co_2Cl_2-(BTDD)	H_2BTDD	353998.53	0.52	184375.30	28
	$CoCl_2$	580.52	0.51		
COF-TpPa	Tp(1,3,5-triformylphloroglucinol)	130472.00	0.56	73084.48	15
	Pa(p-Phenylenediamine)	45.82	0.44		
KMF-1	$Al_2(SO_4)_3 \cdot 18H_2O$	9.84	1.40	124131.92	11
	NaOH	7.83	0.17		
	2,5-pyrroledicarboxylic	427989.00	0.29		
Zr-Fum HT	$ZrCl_4$	78.26	0.15	32.76	12
	Formic acid	6.00	3.04		
	Fumaric acid	12.11	0.23		
AlPO-LTA	H_3PO_4	5.05	0.81	84202.75	9
	$Al(OPri)_3$	16.08	1.45		
	K222	127537.30	0.66		
	HF	4.85	0.15		

Supplementary Table 13. DFT-derived DDEC atomic partial charges for the EMM-8 framework

Al1	2.192	O1	-1.900	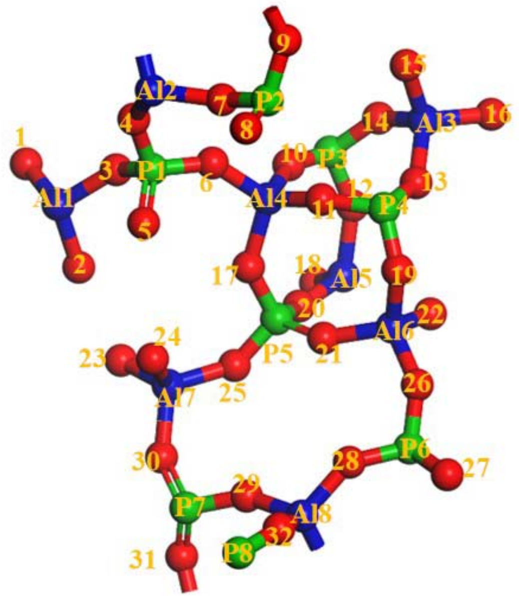			
Al2	2.421	O2	-0.662				
Al3	2.469	O3	-0.798				
Al4	2.268	O4	-1.254				
Al5	2.208	O5	-2.364				
Al6	2.570	O6	-0.734				
Al7	2.370	O7	-0.826				
Al8	2.348	O8	-0.705				
P1	2.997	O9	-0.542	O17	-0.525	O25	-0.413
P2	0.712	O10	-1.687	O18	-1.886	O26	-1.572
P3	2.740	O11	-1.123	O19	-0.839	O27	-0.792
P4	2.010	O12	-0.621	O20	-0.691	O28	-0.820
P5	0.676	O13	-1.172	O21	-0.783	O29	-1.173
P6	3.278	O14	-1.415	O22	-2.488	O30	-1.257
P7	2.020	O15	-1.372	O23	-1.385	O31	-0.830
P8	2.837	O16	-0.451	O24	-1.272	O32	-1.759

Supplementary Table 14. LJ potential parameters for the atoms of the H_2O, and EMM-8.

Elements	σ (Å)	ε (Kcal/mol)	q(e)
Al	2.941	9.043	**List in**
P	4.20	0.200	**Supplementary**
O	3.210	0.228	**Table 13**
OW	3.166	0.155	-0.820
HW	0	0	+0.410

List of Symbols

Latin

Q	energy, kJ·mol^{-1}
m	mass, g
m_{i}	the real-time quality in the adsorption process, mg
m_0	the quality of dehydrated material, mg
$q(P,T)$	amount adsorbed as a weight-ratio, g·g^{-1}
q	dimensionless conversion ratio
q_{i}	water uptake for multiple measurement as a weight-ratio, g·g^{-1}
$\bar{q}$	the average water uptake of multiple measurement, g·g^{-1}
D	particle diameter, mm
D_{M}	diffusion coefficient of water vapor within the particles, cm^2·s^{-1}
A	adsorption potential, kJ·mol^{-1}
W	volume of liquid adsorbed, ml·g^{-1}
ΔW	working capacity, ml·g^{-1}
R	gas constant, J·K^{-1}·mol^{-1}
T	temperature, K
P	pressure, kPa
P/P_0	relative pressure
P_0	saturation pressure of working fluid, kPa
c_p	heat capacity, J·g^{-1}·K^{-1} (J·mol^{-1}·K^{-1})
M_{w}	molar mass, g·mol^{-1}

t	adsorption or desorption time, s

Greek

$\Delta_{ads}H$	entlahpy of adsorption, kJ·mol^{-1}
$\Delta_{vap}H$	entlahpy of evaporation, kJ·mol^{-1}
ρ	liquid density of the adsorbate, g·mL^{-1}
τ_{ads}	characteristic times of adsorption stage, s
τ_{des}	characteristic times of desorption stage, s
$\tau_{0.8ads}$	adsorption time corresponding to the conversion ratio 0.8, s
$\tau_{0.8des}$	desorption time corresponding to the conversion ratio 0.8, s
E	relative uncertainty
μ	uncertainty

Subscripts

ads	adsorption
con	condensation
regen	regeneration
des	desorption
ev	evaporation
max	maximum load of the cycle
min	minimum load of the cycle
sorbent	adsorbent
sorption	adsorption
liq	liquid

0.8	conversion ratio is 0.8
I	point I in **Supplementary Fig. 1**
II	point II in **Supplementary Fig. 1**
III	point III in **Supplementary Fig. 1**
IV	point IV in **Supplementary Fig. 1**
A	type A standard uncertainty
B	type B standard uncertainty

Superscripts

eff	effective
wf	working fluid

Supplementary References

1. de Lange, M. F., Verouden, K. J. F. M., Vlugt, T. J. H., Gascon, J. & Kapteijn, F. Adsorption-driven heat pumps: the potential of metal−organic frameworks. *Chem. Rev.* **115,** 12205-12250 (2015).
2. Yan, J. et al. Adsorption isotherms and kinetics of water vapor on novel adsorbents MIL-101(Cr)@GO with super-high capacity. *Appl. Therm. Eng.* **84,** 118-125
3. Furukawa, H. et al. Water adsorption in porous metal−organic frameworks and related materials. *J. Am. Chem. Soc.* **136,** 4369-4381 (2014).
4. Wang, S. et al. A robust large-pore zirconium carboxylate metal–organic framework for energy-efficient water-sorption-driven refrigeration. *Nat Energy* **3,** 985-993 (2018).
5. Cadiau, A. et al. Design of hydrophilic metal organic framework water adsorbents for heat reallocation. *Adv. Mater.* **27,** 4775-4780 (2015).
6. Reinsch, H. et al. Structures, sorption characteristics and nonlinear optical properties of a new series of highly stable aluminium MOFs. *Chem. Mater.* **25,** 17-26 (2013).
7. Lee, J. S. et al. The porous metal-organic framework CUK-1 for adsorption heat allocation toward green applications of natural refrigerant water. *ACS Appl. Mater. Interfaces.* **11,** 25778-25789 (2019).
8. Yeh, R. L., Ghosh, T. K. & Hines, A. L. Effects of regeneration conditions on the characteristics of water vapor adsorption on silica gel. *J. Chem. Eng. Data.* **37,** 259-261 (1992).

9. Krajnc, A. et al. Superior performance of microporous aluminophosphate with LTA topology in solar-energy storage and heat reallocation. *Adv. Energy Mater.* **7,** 1601815 (2017).

10. Ristic, A., Logar, N. Z., Henninger, S. K. & Kaucic, V. The performance of small-pore microporous aluminophosphates in low-temperature solar energy storage: the structure–property relationship. *Adv. Funct. Mater.* **22,** 1952–1957 (2012).

11. Cho, K. H. et al. Rational design of a robust aluminum metal-organic framework for multi-purpose water-sorption-driven heat allocations. *Nat Commun* **11,** 5112 (2020).

12. Cho, K. H. et al. Defective Zr-fumarate MOFs enable high-efficiency adsorption heat allocations. *ACS Appl. Mater. Interfaces* **13,** 1723-1734 (2021).

13. Lenzen D. et al. A metal–organic framework for efficient water-based ultra-low-temperature-driven cooling. *Nat Commun* **10,** 3025 (2019).

14. Rieth, A. J., Yang, S., Wang, E. N. & Dincã, M. Record atmospheric fresh water capture and heat transfer with a material operating at the water uptake reversibility limit. *ACS Cent. Sci.* **3,** 668-672 (2017).

15. Pérez-Carvajal, J., Boix, G., Imaz, I. & Maspoch, D. The imine-based COF TpPa-1 as an efficient cooling adsorbent that can be regenerated by heat or light. *Adv. Energy Mater.* **9,** 1901535 (2019).

16. Liu, Z. L., Xu, M., Huai, X. L., Huang, C. F. & Lou, L. T. Ionothermal synthesis and characterization of $AlPO_4$ and $AlGaPO_4$ molecular sieves with LTA topology.

Microporous Mesoporous Mater. **305,** 110315 (2020).

17. Kim, Y. D., Thu, K. & Ng, K. C. Adsorption characteristics of water vapor on ferroaluminophosphate for desalination cycle. *Desalination.* **344,** 350-356 (2014).
18. Zhou, L. S., Guan, J. K., Yu, C. L. & Huang, B. C. MnOx Supported on hierarchical SAPO-34 for the low-temperature selective catalytic reduction of NO with NH_3: catalytic activity and SO_2 resistance. *Catalysts.* **11,** 314 (2021).
19. Gordeeva, L. G., Solovyeva, M. V. & Aristov, Y. I. NH2-MIL-125 as a promising material for adsorptive heat transformation and storage. *Energy* **100,** 18-24 (2016).
20. Solovyeva, M. V., Aristov, Y. I. & Gordeeva, L. G. NH2-MIL-125 as promising adsorbent for adsorptive cooling: water adsorption dynamics. *Appl. Therm. Eng.* **116,** 541-548 (2017).
21. Kim, H. et al. Water harvesting from air with metal-organic frameworks powered by natural sunlight. *Science* **356,** 430-434 (2017).
22. Solovyeva, M. V., Gordeeva, L. G., Krieger, T. A. & Aristov, Y. I. MOF-801 as a promising material for adsorption cooling: equilibrium and dynamics of water adsorption. *Energy Convers. Manage.* **174,** 356-363 (2018).
23. Freni, A. et al. SAPO-34 coated adsorbent heat exchanger for adsorption chillers. *Appl. Therm. Eng.* **82,** 1-7 (2015).
24. Lenzen, D. et al. Scalable green synthesis and full-scale test of the metal–organic framework CAU-10-H for use in adsorption-driven chillers. *Adv. Mater.* **30,** 1705869 (2018).
25. Kummer, H. et al. A functional full-scale heat exchanger coated with aluminum

fumarate metal–organic framework for adsorption heat transformation. *Ind. Eng. Chem. Res.* **56,** 8393-8398 (2017).

26. Kim, H. et al. Water harvesting from air with metal-organic frameworks powered by natural sunlight. *Science* **356,** 430-434 (2017).

27. Humphrey, S. M., Chang, J. S., Jhung, S. H., Yoon, J. W. & Wood, P. T. Porous cobalt(II)-organic frameworks with corrugated walls-structurally robust gas-sorption materials. *Angew. Chem.* **119,** 276-279 (2007).

28. Rieth, A. J., Tulchinsky, Y. & Dinca, M. High and reversible ammonia uptake in mesoporous azolate metal-organic frameworks with open Mn, Co, and Ni sites. *J. Am. Chem. Soc.* **138,** 9401-9404 (2016).
